# Development of microsatellite markers and assembly of the plastid genome in *Cistanthe longiscapa* (Montiaceae) based on low-coverage whole genome sequencing

**DOI:** 10.1371/journal.pone.0178402

**Published:** 2017-06-02

**Authors:** Alexandra Stoll, Dörte Harpke, Claudia Schütte, Nadine Stefanczyk, Ronny Brandt, Frank R. Blattner, Dietmar Quandt

**Affiliations:** 1 Centro de Estudios Avanzados en Zonas Áridas (CEAZA)–Universidad La Serena, La Serena, Chile; 2 Nees Institute for Biodiversity of Plants, University of Bonn, Bonn, Germany; 3 Leibniz Institute of Plant Genetics and Crop Plant Research (IPK), Gatersleben, Germany; 4 German Centre of Integrative Biodiversity Research (iDiv) Halle-Jena-Leipzig, Leipzig, Germany; Austrian Federal Research Centre for Forests BFW, AUSTRIA

## Abstract

*Cistanthe longiscapa* is an endemic annual herb and characteristic element of the Chilean Atacama Desert. Principal threats are the destruction of its seed deposits by human activities and reduced germination rates due to the decreasing occurrence of precipitation events. To enable population genetic and phylogeographic analyses in this species we performed paired-end shotgun sequencing (2x100 bp) of genomic DNA on the Illumina HiSeq platform and identified microsatellite (SSR) loci in the resulting sequences. From 29 million quality-filtered read pairs we obtained 549,174 contigs (average length 614 bp; N50 = 904). Searching for SSRs revealed 10,336 loci with microsatellite motifs. Initially, we designed primers for 96 loci, which were tested for PCR amplification on three *C*. *longiscapa* individuals. Successfully amplifying loci were further tested on eight individuals to screen for length variation in the resulting amplicons, and the alleles were exemplarily sequenced to infer the basis for the observed length variation. Finally we arrived at 26 validated SSR loci for population studies in *C*. *longiscapa*, which resulted in 146 bi-allelic SSR markers in our test sample of eight individuals. The genomic sequences were also used to assemble the plastid genome of *C*. *longiscapa*, which provides an additional set of maternally inherited genetic markers.

## Introduction

Microsatellites, a special category of simple sequence repeats (SSRs), are tandemly repeated motifs of one to eight bases that occur in the nuclear genomes of all eukaryotes tested up to now and are, at least for species with larger genomes, quite abundant and evenly dispersed throughout the genome [1,2]. SSR loci are usually characterized by a high degree of length polymorphisms, and are ideal co-dominant markers for population studies. For the analysis of SSRs, specific loci are normally PCR amplified and screened for length differences among individuals. This PCR step constitutes a major drawback of SSR markers, as it involves primers specific for the loci of interest in the species under study. Thus, *de novo* development of SSR markers is necessary for all newly studied taxa. For the Montiaceae species (former Portulacaceae, see [3–6]) *Cistanthe longiscapa* or close relatives up to now no SSR markers are available.

*Cistanthe longiscapa* or “Pata de Guanaco” is an endemic annual of the Atacama Desert, distributed between 25° and 31°S from coastal habitats up to 3800 m elevation in the Andes [7]. It prefers well-drained, sandy soils [8]. After rare winter rainfall *C*. *longiscapa* contributes with its purple-pink flowers and massive bloom in isolated patches to the flowering desert phenomenon. Field observations indicate on-going habitat destruction by off-road activities and tourism especially during flowering times. However, the species seems currently not threatened [9,10].

To enable population genetic analyses in *C*. *longiscapa* we developed SSR markers for this species. By taking advantage of second-generation sequencing technology that easily provides genome-wide sequence information of a species of interest we could avoid the tedious enrichment and cloning steps of traditional SSR development [11]. Therefore, we performed low-coverage shotgun sequencing of the *C*. *longiscapa* whole genome on the Illumina HiSeq platform and identified SSR loci by searching in scaffolds for SSR motifs. For candidate loci PCR primers were designed and afterwards tested by PCR amplification and screening for fragment length polymorphisms among different *Cistanthe* individuals. This resulted in a set of 26 variable SSR loci. Moreover, the sequences provided enough reads derived from the chloroplast to assemble also the entire plastid genome that provides additional maternally inherited marker loci.

## Materials and methods

### Plant material

Plant tissue (whole individuals, fresh 5–20 g each) of *Cistanthe longiscapa* (Barnéoud) Carolin ex Hershkovitz was collected in natural stands of the species in the Atacama Desert (Table 1). As the species is not endangered or protected, no permission was required for its collection at any of the locations sampled in this study; in Chile, no statutory framework governing the collection of unlisted wild plants is currently in force. Plant material was frozen, and stored at -20°C till DNA extraction.

**Table 1:** Used *C*. *longiscapa* individuals for genome sequencing and SSR screening.

Accession ID	Locality	Geographic coordinates	Use ^1^
FN0543	Chile, Los Choros	S 29°15'13"; W 071°25'27"	SL
FN0550	Chile, Los Choros	S 29°15'13"; W 071°25'27"	SL
FN0633	Chile, Sur Quebrada Seca	S 27°35'41"; W 070°51'47"	LT
FN0636	Chile, Sur Quebrada Seca	S 27°35'41"; W 070°51'47"	LT
FN0650	Chile, Camino a Freirina	S 28°22'07"; W 070°49'07"	LT
FN0672	Chile, Camino a Carrizalillo	S 28°57'54"; W 71°10'57"	LT
FN0725	Chile, Pajonales	S 29°17'09"; W 071°01'53"	LT
FN0780	Chile, Punta Colorada	S 29°22'20"; W 070°59'08"	LT
FN0895	Chile, Mamalluca	S 30°01'24"; W 070°41'55"	LT
FN0903	Chile, Mamalluca	S 30°01'24"; W 070°41'55"	LT

^1^ SL = sequencing library; LT = locus testing.

### DNA isolation

*Cistanthe longiscapa* is characterized by high amounts of mucilage within its succulent leaves. To reduce the concentration of these polysaccharides, total genomic DNA was extracted with a modified CTAB protocol [12]. The quality of extracted DNA was checked on 1% agarose gels. DNA concentration was measured using a NanoDrop 2000 photometer (Thermo Scientific). Additionally, the two individuals used for the sequencing library were measured with the Qubit DNA Assay Kit in a Qubit 2.0 Flurometer (Life Technologies).

### DNA library preparation and sequencing

As the obtained DNA amounts per individual were rather low, we combined DNAs of two *C*. *longiscapa* individuals (Table 1) for construction of the sequencing library. In total 0.166 μg genomic DNA was used as input material for the following steps. Library preparation was carried out as described by Meyer and Kirchner [13]. Briefly, DNA was covarized to generate fragments of on average 300–400 base pair (bp) length followed by adaptor and barcode ligation. The library was size-selected with a SYBR Gold stained electrophoresis gel. Fragment size distribution and DNA concentration were evaluated on an Agilent BioAnalyzer High Sensitivity DNA Chip and using the Qubit DNA Assay Kit in a Qubit 2.0 Flurometer (Life Technologies). Finally the DNA concentration of the library was checked by a quantitative PCR run. Cluster generation on Illumina cBot and paired-end sequencing (2x100 bp) on the Illumina HiSeq 2000 platform followed Illumina’s recommendation and included 1% Illumina PhiX library as internal control.

### Data filtering and de novo assembly

As only about one fifth of a HiSeq lane was used to generate the *C*. *longiscapa* sequences, sequence reads were initially sorted according to their barcodes to separate them from the other materials sequenced in parallel. Before genome assembly the 92 million obtained raw sequencing reads (46 million pairs) were quality checked and over-represented, i.e. clonal reads were detected with FastQC [14]. Quality trimming (minimum length of 75 bp and Phred score of at least 15) and adaptor sequence removing was done in Cutadapt v0.11.1 [15]. *De novo* genome assembly of the 29 million quality-filtered read pairs was performed by Clc v4.3.0 (CLC bio) followed by scaffolding with sspace v3.0 [16] to improve the assembly. NCBI Blast searches were used to check for bacterial contaminations in the sequence reads.

Plastid scaffolds were identified using Blast searches. Scaffolds were mapped against the plastid chromosome of *Beta vulgaris* (GenBank accession number KR230391) and *Haloxylon persicum* (KF534479). Gaps were filled using GapFiller v1.10 [17]. Proper pairing of reads was checked by mapping the original reads against the obtained *C*. *longiscapa* plastid chromosome using Bowtie2 v2.2.4 [18] and manual examining by visualization using SAMtools v1.2 [19]. Additional Sanger sequencing was performed for the IR/SSU boundaries (see below). Therefore, universal angiosperm primers were designed using different available angiosperm chloroplast genomes in combination with already established primers (compare Table 2). In addition, minor sequence parts of two genes (*ycf*2 and *trn*A) that did not pass the quality checks after mapping where confirmed by Sanger sequencing (compare Table 2). Amplification of the plastid regions were performed in a 25 μL reaction volume containing 0.75 U DNA polymerase, (GoTaq, Promega), 1 x buffer, 0.2 mM of each dNTP, 2.5 mM MgCl_2_, 20 pmol of each amplification primer, and about 10 ng of total DNA. The amplifications were performed in a Mastercycler Pro (Eppendorf) with the following PCR protocol: 2 min initial denaturation at 94°C and 35 cycles of 30 s at 95°C, 60 s at 52°C, 60 s at 72°C, followed by a final extension for 10 min at 72°C. Amplified products were gel cleaned using spin filter columns (NucleoSpin Gel, Macherey-Nagel) following the manufacturer’s protocol and sequenced by GATC Biotech (www.gatc-biotech.com). Annotation was performed using CpGAVAS [20] and edited manually guided by the *Lindenbergia philippensis* (NC022859) annotation [21]. The map of the plastid chromosome was generated using GenomeVx [22]

**Table 2:** Primers for the validation of the IR boundaries. ‘*ycf*1’ = pseudogene.

Region	Code	O	Primer Sequence
LSC-IR_A_ (*rps*19-*trn*I)	cp259	F	TAATAAATGATTCGCTACAAAAGG
	cp260	R	TCTATTGGAATTGGCTCTGTATC
IR_A_-SSC (‘*ycf*1’-*ndh*F)	cp211	F	ACCAAGTTCAATGTTAGCGAGATTAGTC^§^
	cp213	R	GTCTCAATTGGGTTATATGATG^#^
SSC-IR_B_ (*ycf*1)	cp262	F	TTGTATGACCABCGAGGAACTTTTTTAC
	cp211	R	ACCAAGTTCAATGTTAGCGAGATTAGTC^§^
*ycf*2	cp263	F	CTCACTATTTCTTAGATTCATG
	cp264	R	TTAACCATTTCTTTATTTTCCG
*trn*A	cp265	F	CAACGGAGAGTTGTATGCTG
	cp266	R	GGTCCTCTTCCCCATTACTT

^§^Jansen [23]

^#^ Olmstead & Sweere [24].

### Search for SSR loci and primer design

Potential SSR markers were detected in the scaffolds of the assembled nuclear genome using the Misa tool [25]. We searched for SSRs with motifs ranging from di- to hexa-nucleotides. The minimum number of repeat units was set as following: ten for di-, eight for tri-, seven for tetra-, six for penta- and five for hexa-nucleotide motifs. Raw reads for the SSR contigs and proper pairing of reads was cross checked aligning the raw reads to the SSR loci using Bowtie2 v2.2.4 [18] and manual examining by visualization using SAMtools v1.2 [19]. Primer pairs were designed using Primer3 [26] with default parameters.

### Assessment of SSR polymorphisms

We selected 96 loci (74 tri-, 19 tetra-, and 3 penta-nucleotide repeat motifs) from the nucleus for which primer pairs were synthesized (SigmaAldrich). Our selection criteria were (*i*) high motif repeat number per locus to increase the chance to find variation, (*ii*) avoiding repeat motifs that tend to form strong secondary structures (e.g., GC/CG, TAA/ATT) and are therefore hard to amplify and/or score, (*iii*) long enough and suitable regions flanking the SSR motifs to place PCR primers in, and (*iv*) low to medium read coverage in the sequences of the selected loci to avoid targeting SSRs within repetitive regions of the genome. Amplification success was tested on three *C*. *longiscapa* individuals, of which two individuals came from the same and one from a geographically widely separated population. Polymerase chain reactions (PCR) were carried out in 25 μL reaction volumes containing 1 U *Taq* DNA polymerase (GoTaq, Promega), 0.2 mM of each dNTP, 1 x buffer, 2.0 mM MgCl_2_ and 10 pmol of each amplification primer. The amplification profile was composed as follows: 2 min at 94°C, 35 cycles of 30 s at 94°C, 60 s at 55°C, 60 s at 72°C, followed by a final extension step of 10 min at 72°C.

For loci passing this test, products were subsequently cloned using the pGEM-T Easy Kit (Promega), and clones were sequenced at Macrogen (Korea). Lengths and sequences of 49% of the alleles were confirmed through cloning and sequencing. Among the 73 sequenced loci an allelic diversity of 23% was detected. Quality of the sequence reads was assessed using PhyDE [27], and sequences were aligned with the vector and primer sequences being removed. This enabled us to detect polymorphic SSR markers and to directly infer the allelic state of them, i.e. to see if the SSR motif causes the size difference or if length mutations in the flanking regions are present. Only for loci with length variability fluorescent-labeled forward primers were ordered and fragment length polymorphisms among eight *C*. *longiscapa* genotypes from six populations (Table 1) evaluated using the service provided by Macrogen (Korea). Fragment data were analyzed in Geneious R9 [28], the statistical analyses of allelic diversity were performed using GenAlEx [29].

## Results

### Illumina shotgun sequencing

Paired-end sequencing yielded about 46 million reads from either end of the DNA fragments, resulting in a total amount of about 9 Gb. After quality assessment and data filtering, 29 million clean read pairs were classified as high quality reads (63%) and used for further analysis.

### Genome assembly

The high-quality reads of lengths between 80 and 100 bp were used for genome assembly. They resulted in 549,174 contigs with a minimum length of 200 bp, a maximum length of 33,958 bp, a N50 of 742 bp and an average length of 570 bp. Scaffolding increased the N50 to 904 bp, the average scaffold size of the 489,721 scaffolds was 641 bp with a maximum scaffold size of 85,841 bp.

**Fig 1:**
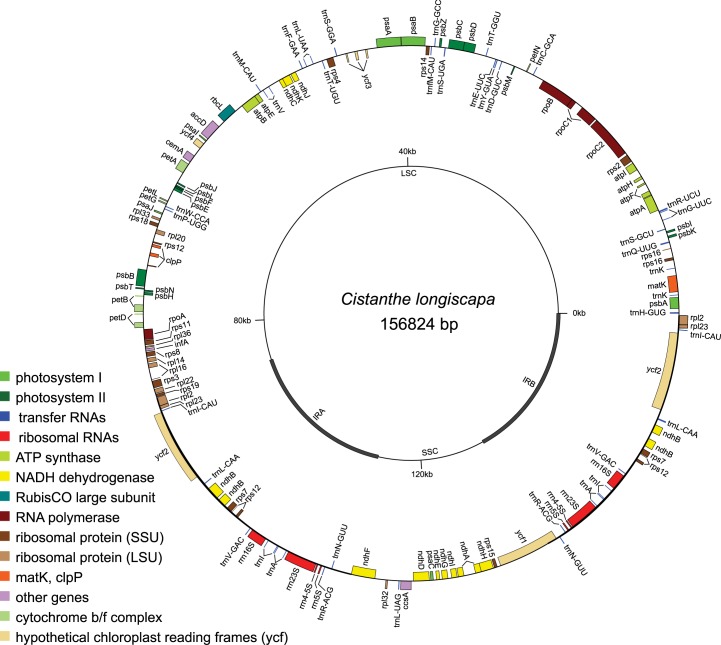
Schematic representation of the annotated *C*. *longiscapa* plastid genome.

The assembled plastid genome of *C*. *longiscapa* contained contigs, which were generally present with very high sequence coverage (500-2000-fold). The length of the genome is 156,824 bp, consisting of 86,715 bp for the large single-copy region, 18,363 bp for the small single-copy region, and twice 25,873 bp of sequences belonging to the two inverted-repeat (IR) regions (compare Table 3). IR boundaries and questionable positions in *ycf*2 and *trn*A could be validated by Sanger sequencing as outlined above. Two polymorphic sites were detected in the non-coding parts of the SSC, one in the *rpl*23-*trn*L intergenic spacer (IGS), the other one in the *ndh*A intron. The chromosomal architecture mirrors the typical structure found in angiosperms [30], with the exception of group II intron loss in *rpl2*. In total we found 79 protein coding genes, 30 tRNAs and 4 rRNAs. The genome annotation is shown in Fig 1, the sequence is available from GenBank through accession number KX928992.

**Table 3:** Summary of plastome features in *C*. *longiscapa* and comparison to other Caryophyllales and *Lindenbergia philippensis* (Orobanchaceae, Lamiales).

	*Cistanthe longiscapa*	*Haloxylon persicum*	*Beta vulgaris*	*Lindenbergia philippensis*
Total size	156,778 bp	151,586 bp	149,635 bp	155,103 bp
LSC length	86,715 bp	84,217 bp	83,057 bp	85,594 bp
IR length	25,850 bp	24,177 bp	24,439 bp	25,812 bp
SSC length	18,363 bp	19,015 bp	17,701 bp	17,885 bp
Total GC content	36.7%	36.6%	36.4%	37.7%
GC content LSC	34.5%	34.5%	34.1%	35.8%
GC content IR	42.7%	43.0%	42.2%	43.2%
GC content SSC	30.3%	29.7%	29.2%	31.9%
Total number of genes	113	112	113	113
Protein coding genes	79	78	79	79
tRNA	30	30	30	30
rRNA	4	4	4	4
Genes with introns	17	18	18	18

### Identification of SSR loci

After Misa analysis, a total of 10,336 potential SSR loci fitting our search criteria were identified in the nuclear genome. Of these, the most frequently detected SSR sequences consisted of di-nucleotide repeats with 80.3% of abundance, tri-nucleotide repeats (13.2%), followed by hexa- (2.4%), tetra- (1.2%), and penta-nucleotide repeats (0.6%). Compound SSRs, i.e. two SSR stretches in close proximity, were found in 2.3% of all cases. Repeat numbers of scored SSR motifs were in a range from five (lower cut-off) to 31. PCR amplification primers for the nucleotide repeat loci were tested through PCR amplifications in three individuals. Of the 35 reliably amplifying loci, 26 showed length variation in the test sets of eight *C*. *longiscapa* individuals. Primer sequences together with range parameters and detected allele numbers for these loci are provided in Table 4. In contrast to the abundant nuclear microsatellites, the plastome offered only homonucleotide regions.

**Table 4:** Properties of the selected SSR loci.

Locus	Primers (5’ -> 3’)	Repeat Motif	ASR	N_A_	H_O_-H_E_	GenBank Acc. No.
Pool 1						
CistLc1	F: CAGTGATTGTTTGGCATTGG	(ATGT)_6-23_	167–207 (6FAM)	6	0.333*-0.167	LT593975–79
	R: GCAGATCCGACTCTTTGGTG					
CistLc2	F: TCAAGTCGCTGACACGGATA	(TC)_5-22_ +(CTGT)_0-8_ +(TC)_0-15_	243–279 (6FAM)	11	0.667–0.458	LT593980–85
	R: TTCATTTGGATGCAAGTTTCC					
CistLc3	F: ACTTTGGGTGCTTGGATGTC	(ATGT)_5-19_	140–156 (VIC)	6	0.167**-0.125	LT593986–89
	R: TTGAACTCTCTTGAACTACAATCGTT					
CistLc4	F: CCCCAAACCAAAAGACAAGA	(TCA)_13-17_	183–189 (VIC)	3	0.083**-0.146	LT593990–93
	R: GGGGACATGGGAGTATGATG					
CistLc5	F: TGTTGGTTTTCTGGGGAGAG	(TGA)_6-12_	174–195 (NED)	7	0.583*-0.354	LT593994–96
	R: CGCCAAACCAGGTCTTCTTA					
CistLc6	F: CGATGCATCCCATTCTCTCT	(TCTA)_5-11_	149–173 (PET)	6	0.333*-0.188	LT593997–LT594001
	R: CGGGAGCTATGGCTTAAAGA					
Pool 2						
CistLc7	F: TTGTGGCATATTGTCGGTGT	(TAGA)_3-8_	158–173 (6FAM)	5	0.167**-0.167	LT594002–05
	R: AGGTCCCGTTGGGAATAATG					
CistLc8	F: TGGATGAGTTTTGCGTAGGA	(TATC)_4-11_	214–234 (6FAM)	5	0.250*-0.146	LT594006–09
	R: GACCCATATGTGCTTCTCCAA					
CistLc9	F: TCTGTGATCCCAGGACCTTC	(ATC)_8-13_	187–202 (VIC)	5	0.167*-0.167	LT594010–13
	R: ATCGGGGGTAGCTTCAAGAC					
CistLc10	F: CTCGAATCTTATCGCCCAAA	(GAT)_9-15_	178–193 (NED)	6	0.500–0.271	LT594014–18
	R: TGCATCTTCCTTCCTTGCTT					
CistLc11	F: GAATAAAATCAGGGCCGTTG	(ATG)_4-10_	131–152 (PET)	6	0.750–0.458	LT594019–24
	R: GCACTTGCAGCTCTGTACCA					
Pool 3						
CistLc12	F: CATCAACGAATCACCAATGC	(CTAT)_3-10_	135–155 (6FAM)	6	0.333–0.292	LT594025–29
	R: GATGGAAAGAGAGCGCAAAT					
CistLc13	F: TCAAAAAGCAAAATACTTAACTTCC	(ACAT)_5-11_	182–198 (6FAM)	6	0.500–0.292	LT594030–35
	R: TCCTTGTTTTGAGGTTCATCG					
CistLc14	F: GGGTTGAGCTTGATTTGGAA	(GAT)_9-12_	145–157 (VIC)	4	0.500*-0.250	LT594036–40
	R: CAGCACTCGGAGTTTCACCT					
CistLc15	F: TCTGCCACTATAGCATAAGCAG	(ATC)_7-12_	179–200 (NED)	4	0.333–0.208	LT594041–43
	R: AAAACACGGGCTCATTCATT					
CistLc16	F: CCTCTCCAAACCCACTTCAA	(TGA)_8-17_	169–184 (PET)	5	0.500–0.250	LT594044–47
	R: AGGTTTCGAACATTCCATCTG					
Pool 4						
CistLc17	F: ATTTTCACTTGGGTGCCTTG	(TGA)_10-13_	136–157 (6FAM)	4	0.333–0.208	LT594048–51
	R: TCCATCACATCATCCTCGTC					
CistLc18	F: AAGGCATCCCTTCTGTCCTT	(TTAAG)_5-8_	225–240 (6FAM)	4	0.500*-0.250	LT594052–55
	R: GCCTAAAAGCAGTACCGATTCA					
CistLc19	F: CCAGAGGAGGAGGGGTTAAA	(CAT)_10-14_	180–206 (VIC)	9	0.500*-0.271	LT594056–59
	R: TGGAGGGTGAGAATTCAGAG					
CistLc20	F: AGTATGTGGGGCACTTTTGC	(TAC)_6-9_	154–166 (NED)	5	0.500–0.271	LT594060–64
	R: TCCCATTCATCAATTAAGGTATCA					
CistLc21	F. CAATTTCTGGTGTTGCTGATCT	(GAT)_1-11_	165–187 (PET)	6	0.500*-0.333	LT594065–70
	R: TGATCCCCATGAAAATCCTG					
Pool 5						
CistLc22	F: CGAAACTCGCTCCATTTCTC	(GAG)_4-9_	154–161 (6FAM)	4	0.500–0.271	LT594071–74
	R: CCAAGGAGTTGCAAACACAA					
CistLc23	F: TCCGAGGAACTTTCGCTAGA	(TATAG)_8-15_	201–261 (6FAM)	6	0.000***-0.167	LT594075–80
	R: CCGCATCAAAGACAGATTCA					
CistLc24	F: TGCAGAAGAAGAGGGTGATTG	(ATC)_5-9_	186–199 (VIC)	5	0.333–0.188	LT594081–86
	R: CCTCACTCCCAGAGCCATAG					
CistLc25	F: CGCAAATGTCCCCAGTATCT	(GAT)_8-15_	167–200 (NED)	6	0.333–0.271	LT594087–89
	R: CATTCAACCTCTTTGCGTCA					
CistLc26	F: GCTGCCCGACTAATTTTGAA	(GAT)_3-14_	142–160 (PET)	6	0.583–0.333	LT594090–94
	R: CAGACAAGCCAGATGCATGA					

Pool numbers refer to the pooling strategy in SSR analysis. ASR = amplicon size range, () including proposed dye strategy for the pools, 6FAM/VIC/NED/PET = standard dyes for multiplexing, f, N_A_ = Number of alleles, H_O_ = observed heterozygosity, H_E_ = expected heterozygosity.

Asterisks indicate different significance levels between the observed and expected heterozygosity under Hardy–Weinberg equilibrium

* P<0.05

** P<0.01

*** P<0.001.

### Sequencing of the cloned SSR alleles

Cloning and sequencing of the 35 reliably amplified loci confirmed the contigs retrieved from the initial shotgun sequencing. Although the expected SSR motifs were present in all loci, six loci (17%) were excluded from the marker panel due to length mutations adjacent to the SSR motif. For two loci only a small fraction of the sequenced clones were similar to the initial shotgun sequences. They were therefore excluded, as PCR primers seemed not to be locus specific. Finally, one locus was excluded due to an imperfect SSR pattern, leaving 26 loci for the microsatellite test run with the following proportion of nucleotide repeats: 61.5% tri-, 26.9% tetra-, and 7.7% penta-nucleotide repeats plus 3.8% compound (di- plus tetra-nucleotide repeat) motifs. GenBank sequence accession numbers are given in Table 4. According to the PCR amplicon size range of the selected microsatellite loci we propose a multiplexing strategy based on five pools using 6FAM/VIC/NED/PET as standard dyes in fluorescent detection, as indicated in Table 4.

### Detected length variation in chosen SSR loci

Fragment analyses revealed on average six alleles per locus (min = 3; max = 11). The observed heterozygosity (*H*_O_) per locus ranged from 0.000 to 0.750, while the expected heterozygosity (*H*_E_) varied from 0.125 to 0.458. Half of the 26 polymorphic loci departed significantly from the Hardy–Weinberg equilibrium.

## Discussion

To arrive at a set of SSR markers for population studies in *C*. *longiscapa* we used sequences derived from a shotgun sequencing approach of genomic DNA. We did not enrich the sequencing library for SSR motifs (e.g., [31]) prior to sequencing, as the high amount of SSR loci in plant genomes should result, even in untreated genomic DNA, in a sufficient number of useful regions to successfully develop SSR markers. Moreover, this speeds up the development procedure, and the comparatively low costs for next-generation sequencing easily compensates the reduced ratio of SSR loci vs. non-SSR regions within the resulting sequences.

We here used mainly loci derived from scaffolds with 3x-10x sequence coverage. We avoided including SSR loci derived from contigs and scaffolds with very high sequencing coverage (>15x), as they likely stem from repetitive parts of the genome. They bear the risk to include SSR loci that occur as paralogs with multiple copies within a genome. Accordingly, the final set of our SSR loci did not produce fragment patterns indicative of multilocus data, i.e. amplicons resulting from the presence of two or more paralogous loci detected by a single PCR primer pair. The DNA sequence of scaffolds with such low sequencing coverage might include some uncertain sequence positions, which could result in a lower number of reliably amplifying SSRs during the test of the 96 initially chosen loci due to primer mismatches. However, the initial PCR step in our development procedure selects against such loci, as unreliably amplifying loci were not included in the next steps of locus evaluation. For us, initial omission of potentially multicopy loci, which might produce analysis problems in a later stage of the project, seems preferable over retaining a higher number of SSR loci throughout the early stages of SSR development.

The interpretation of microsatellite data faces different kinds of errors [32]. These are due to (*i*) parallel mutations in the SSR motif in different individuals resulting independently in the same fragment size, (*ii*) length mutations in the flanking region of the SSR motif that influence fragment lengths of some individuals but are thought to stem from repeat number differences of the SSR, and (*iii*) base substitutions creating new alleles without changing fragment lengths. While it is not possible to detect errors of the first class, sequencing of the allelic diversity found in a species can possibly reduce problems derived from the other two. Thus, we sequenced SSR alleles during the validation of the SSR loci and excluded about one quarter of the reliably amplifying loci (9 out of 35) from our marker set. This can, of course, not prevent that, with additional individuals included, some of these errors might occur. It helped, however, that obviously problematic loci were excluded from further analyses and should provide an overall better set of SSR markers.

Although 26 SSR loci (27% of the initially selected 96 loci) were validated here, we explored only a very small fraction of potentially variable SSR sites in the genome of *C*. *longiscapa*. Whole genome shotgun sequencing, even when performed with rather low genomic coverage (here using ~20% of a HiSeq lane), detected more than 10,000 potentially useful SSR loci. With less stringent selection conditions (looking also for lower repeat numbers within SSR motifs) even more than 95,000 SSR loci were reported [33]. Thus, by using next-generation sequencing for the detection of SSR loci it now is possible to scale the amount of available SSR markers in a wide range, depending on the questions at hand. For population genetic analyses in *C*. *longiscapa*, 26 loci seem to provide a good resource to infer differentiation among populations. In case of genomic mapping studies a much higher amount of variable marker would be necessary to densely cover a genome but could easily be derived from the initial set of thousands of SSR motifs found in the genome of the species. The now much faster, easier and cheaper procedure of SSR development in comparison to traditional sequence enrichment and cloning approaches [11] allows to easily design variable genetic markers for nearly every species of interest. *De novo* development of SSR loci should also be superior to marker transfer from closely related species, as this often result in the use of suboptimal, i.e. less variable loci in comparison to specifically designed SSRs [34].

Genomic shotgun sequencing resulted, in addition to the nuclear SSR loci, in the completely assembled plastid genome of *C*. *longiscapa* (Fig 1). This genome contains 86 regions of mononucleotide repeats (10–23 A/T repeats), representing the simplest class of microsatellite motifs. As we used two individuals of *C*. *longiscapa* to construct the sequencing library, we found some positions in the plastid sequence, which were inferred to already represent polymorphic characters like in the *rpl*23-*trn*L IGS or the *ndh*A intron. These parts of the genome can be used to evaluate maternally inherited genomic diversity for phylogeographic studies (e.g., [35]) in the species. In our case, both individuals used for sequencing were derived from the same population. Using instead individuals from geographically distant parts of the distribution area might even increase the number of detectable polymorphic sites within the plastid genome.
